# Effects of Prenatal Stress on Behavior, Cognition, and Psychopathology: A Comprehensive Review

**DOI:** 10.7759/cureus.47044

**Published:** 2023-10-14

**Authors:** Aniket Jagtap, Balasaheb Jagtap, Rajlaxmi Jagtap, Yashwant Lamture, Kavita Gomase

**Affiliations:** 1 Surgery, Jawaharlal Nehru Medical College, Datta Meghe Institute of Higher Education and Research, Wardha, IND; 2 Medical Intern, Annasaheb Chaudaman Patil Memorial Medical College, Dhule, IND; 3 Medical Student, Bharti Vidyapeeth Deemed University, Sangali, IND; 4 Surgery, Jawaharlal Nehru Medical College, Datta Meghe Institute of Higher Education and Research, wardha, IND; 5 Obstetric and Gynecological Nursing, Srimati Radhikabai Meghe Memorial College of Nursing, Datta Meghe Institute of Higher Education and Research, Wardha, IND

**Keywords:** interventions, mechanisms, psychopathology, cognition, behavior, prenatal stress

## Abstract

Prenatal stress is increasingly recognized as a significant factor impacting an individual's life from the beginning. This comprehensive review explores the intricate relationship between prenatal stress and its effects on behaviour, cognition, and psychopathology. Key findings reveal that prenatal stress can lead to a wide range of adverse outcomes in offspring, including neurodevelopmental disorders, emotional dysregulation, cognitive deficits, mood disorders, and an increased risk of psychopathological conditions. These effects' mechanisms involve epigenetic modifications, hypothalamic-pituitary-adrenal (HPA) axis dysregulation, neurodevelopmental alterations, inflammatory processes, and changes in brain structure and function. Moreover, moderating factors such as maternal stress levels, maternal mental health, socioeconomic status, social support, and early-life adversity can significantly influence the impact of prenatal stress. The review also discusses intervention and prevention strategies, emphasizing the importance of prenatal stress reduction programs, maternal mental health support, nutritional interventions, and targeted early interventions for at-risk populations. These findings have substantial implications for public health and clinical practice, highlighting the need for a holistic approach to prenatal care that prioritizes maternal well-being and mitigates the lasting effects of prenatal stress. Addressing this critical issue promises healthier generations and stronger communities in the future.

## Introduction and background

Prenatal stress, a phenomenon characterized by elevated maternal stress levels during pregnancy, has garnered significant attention in the fields of psychology, neuroscience, and public health. The recognition of the potential long-lasting impact of prenatal stress on the developing fetus and future offspring has prompted extensive research in recent years. This comprehensive review aims to provide a thorough exploration of the multifaceted relationship between prenatal stress and its effects on behavior, cognition, and psychopathology in individuals across the lifespan [1-4].

Prenatal stress refers to the physiological and psychological stress experienced by expectant mothers during pregnancy. It encompasses a wide range of stressors, both chronic and acute, that can significantly influence the intrauterine environment. These stressors may include maternal anxiety, depression, exposure to traumatic events, socioeconomic challenges, and environmental factors such as pollution and noise. Prenatal stress is a complex construct, with various contributing factors that can interact and manifest in diverse ways [5]. Understanding prenatal stress necessitates not only an examination of maternal experiences but also an exploration of the mechanisms by which these stressors can affect the developing fetus. This includes consideration of hormonal changes, epigenetic modifications, and alterations in placental function, which can all play critical roles in transmitting the effects of maternal stress to the developing fetus [6].

The importance of studying prenatal stress lies in its potential to shape the health and well-being of future generations. Research in this area has expanded our understanding of how early-life experiences can have lasting effects on behavior, cognition, and mental health outcomes. Prenatal stress is not a solitary event but rather a dynamic process that can set in motion a cascade of biological and psychological responses that influence an individual's trajectory throughout life [7]. Moreover, the study of prenatal stress is of paramount significance for public health and clinical practice. Identifying the links between maternal stress during pregnancy and adverse outcomes in offspring can inform targeted interventions and prevention strategies. By recognizing the potentially modifiable factors that contribute to prenatal stress and its consequences, healthcare providers and policymakers can work toward improving the well-being of both expectant mothers and their future children [8].

The primary purpose of this comprehensive review is to synthesize existing knowledge of the impact of prenatal stress on behavior, cognition, and psychopathology. By collating and critically evaluating the findings from a diverse array of studies, we aim to provide a comprehensive overview of the current state of research in this field. This review will explore the various dimensions of prenatal stress, ranging from its definitions and theoretical underpinnings to the mechanisms that mediate its effects.

## Review

Theoretical framework

Stress and its Physiological Effects During Pregnancy

Stress during pregnancy transcends being purely psychological; it encompasses profound physiological implications for the expectant mother and the developing fetus. Maternal stress triggers a complex response within the body known as the hypothalamic-pituitary-adrenal (HPA) axis activation. This physiological cascade results in the elevated production of stress hormones, most notably cortisol. This is particularly significant because cortisol, a pivotal stress hormone, is not confined to the maternal bloodstream; it can readily cross the placental barrier. This cross-boundary transmission exposes the developing fetus to stress signals circulating in the maternal bloodstream [9].

The ramifications of this exposure are multifaceted. Elevated cortisol levels in the fetal environment can perturb the intricate orchestration of fetal development, including the growth and maturation of vital organs, notably the brain. The developing fetal brain, a remarkable and highly plastic structure, is susceptible to cortisol influences. Prenatal stress can disrupt the normal trajectory of brain development, potentially leading to structural and functional alterations. These alterations may manifest later in life as behavioural, cognitive, or emotional consequences for the offspring [10].

Fetal Programming Hypothesis

The fetal programming hypothesis represents a groundbreaking concept that fundamentally alters our understanding of how prenatal experiences can have profound, lifelong consequences. This hypothesis posits that the environment in which a developing fetus gestates, including factors like prenatal stress, possesses the remarkable ability to "program" the fetus for future health and disease outcomes. It suggests that the intrauterine environment is a crucial determinant in shaping an individual's susceptibility to various health conditions that may manifest later in life [11].

Of particular significance is the role of prenatal stress within this hypothesis. Prenatal stress, recognized as a potent environmental factor, emerges as a pivotal player in the programming of the fetal stress response system. This programming involves intricate physiological and molecular changes that can persist long after birth. For instance, exposure to maternal stress during pregnancy can mold the development and functioning of the fetal HPA axis, which regulates the stress response [12].

The implications of this fetal programming are extensive, extending well into adulthood. Prenatal stress can result in alterations in stress reactivity, potentially rendering individuals more susceptible to heightened stress responses throughout their lives. Furthermore, these early programming effects may contribute to an increased vulnerability to mental health disorders, such as anxiety, depression, and mood disorders, in adulthood [13].

The fetal programming hypothesis challenges the traditional paradigm separating prenatal and postnatal health influences. It underscores the enduring impact of prenatal experiences. It emphasizes the need for a holistic approach to healthcare that considers the prenatal environment as a critical determinant of long-term well-being. Understanding how prenatal stress fits within this paradigm provides valuable insights into the origins of health and disease. It paves the way for innovative strategies to promote healthier outcomes for future generations [14].

Role of Maternal and Environmental Factors

Acknowledging the multifaceted nature of prenatal stress, it becomes evident that this phenomenon does not act in isolation but within a complex web of maternal and environmental factors. These factors, intricately interwoven with prenatal stress, play pivotal roles in shaping the developmental landscape of the unborn child. Exploring these influences unveils the nuanced dynamics at play [4]. First and foremost, maternal factors wield significant influence. The mental health of the expectant mother emerges as a critical player in this intricate equation. Maternal mental health, encompassing conditions like depression, anxiety, and stress, coexists with prenatal stress and interacts synergistically. The co-occurrence of maternal stress and mental health conditions can amplify their individual effects, potentially intensifying the impact on the developing fetus [9].

Socioeconomic status (SES), another influential maternal factor, is closely intertwined with prenatal stress. Mothers from disadvantaged socioeconomic backgrounds often grapple with heightened stressors, including financial instability and limited access to healthcare. These stressors can synergize with prenatal stress, compounding the challenges the mother faces and her developing child [15]. Lifestyle behaviors, comprising maternal nutrition and substance use, further mold the prenatal environment. Inadequate maternal nutrition can deprive the fetus of essential nutrients for healthy development. At the same time, substance use, such as tobacco, alcohol, or illicit drugs, can pose direct threats to fetal well-being. These factors augment prenatal stress and present independent risks [16].

On the environmental front, external factors contribute to the intricate prenatal milieu. Exposure to toxins and environmental pollutants can introduce additional stressors, potentially exacerbating the effects of prenatal stress. Conversely, access to robust social support networks can act as a protective buffer, attenuating the impact of maternal stress and promoting healthier outcomes [17]. Appreciating the complexities of these interactions is pivotal in unraveling the enigma of how prenatal stress exerts its influence. It highlights the multifaceted nature of prenatal development, where many factors converge to shape an unborn child's life trajectory. Understanding these intricate dynamics enriches our comprehension of prenatal stress and underscores the necessity of holistic, multidisciplinary approaches to prenatal care and support [18].

Developmental Origins of Health and Disease (DOHaD) Perspective

The DOHaD perspective revolutionizes our understanding of prenatal stress's profound and enduring impact. Essentially, it extends our gaze beyond immediate health outcomes and invites us to contemplate the far-reaching consequences that prenatal experiences can imprint across the entire lifespan. Embracing this perspective reshapes our approach to prenatal stress research, emphasizing the importance of long-term health trajectories and the potential emergence of chronic diseases in adulthood [19].

Under the guidance of the DOHaD perspective, research endeavors to peel back the layers of complexity that shroud the mechanisms through which prenatal experiences cast their long shadows. These mechanisms span multiple domains, from epigenetics to developmental programming, and encompass intricate molecular and physiological processes. Central to this exploration is the understanding that the environment in which a fetus gestates is far from static; it possesses the remarkable capacity to orchestrate developmental adaptations in response to prevailing conditions [19].

Within the DOHaD framework, prenatal stress emerges as a potent modulator of these adaptations. It is not merely an isolated event but a critical determinant that can recalibrate the developing organism's trajectory. Prenatal stress can influence the epigenetic marks that adorn genes, potentially altering their expression in ways that resonate throughout life. It can sculpt the developing fetal systems, including those responsible for stress regulation, influencing an individual's stress reactivity well into adulthood [20].

Perhaps most strikingly, research guided by the DOHaD perspective has unveiled a concerning possibility: prenatal stress may lay the groundwork for emerging chronic diseases in adulthood. This includes conditions such as cardiovascular disease, diabetes, and metabolic disorders, which may stem from the early-life adaptations programmed by prenatal stress. It thus accentuates the need for a comprehensive, lifelong approach to health that begins not in adulthood but in the womb [21].

Prenatal stress and behavior

Behavioral Outcomes in Offspring

Neurodevelopmental disorders: Prenatal stress casts a shadow over the developing child's behavioral health, with associations identified between maternal stress during pregnancy and an elevated risk of neurodevelopmental disorders in offspring. Conditions such as autism spectrum disorder (ASD) and attention deficit hyperactivity disorder (ADHD) have been closely examined in this context. Research suggests that prenatal stress may be a precursor by exposing the developing fetus to maternal stress hormones and inflammatory responses. These exposures can disrupt the intricately choreographed processes of brain development, contributing to alterations in neural circuitry and increasing vulnerability to these neurodevelopmental conditions [22,23].

Emotional regulation: The effects of prenatal stress extend to emotional regulation in offspring. Fetal exposure to maternal stress can result in disturbances in the development of emotional regulation mechanisms, leading to difficulties in managing emotions and coping with stressors. This can manifest as heightened anxiety, depression, and emotional dysregulation later in life. The profound impact on emotional regulation can potentially set the stage for enduring mental health challenges, emphasizing the importance of addressing prenatal stress as a critical determinant of emotional well-being [4].

Temperament and personality traits: Emerging evidence suggests that prenatal stress can subtly influence an individual's temperament and personality traits. Studies have illuminated connections between prenatal stress exposure and the emergence of specific traits, including heightened shyness, introversion, and behavioral inhibition in children. These alterations in temperament may have far-reaching effects, shaping an individual's social and emotional development trajectory. By modulating an individual's predisposition to certain personality traits, prenatal stress underscores its intricate role in shaping the behavioral landscape of future generations [24].

Potential Mechanisms Underlying Prenatal Stress and Behavior

Epigenetic modifications: Prenatal stress can initiate epigenetic modifications in the developing fetus, leading to changes in gene expression patterns within the brain. These epigenetic alterations can endure across the lifespan and play a pivotal role in shaping neural circuits that underlie behavior and emotional regulation. These modifications represent a lasting imprint of prenatal stress on the developing brain [25].

HPA axis dysregulation: Prenatal stress can disrupt the functioning of the HPA axis in offspring. Dysregulation of the HPA axis can lead to long-term alterations in stress hormone regulation. Such dysregulation has been closely associated with mood disorders, anxiety, and altered stress responses, directly linking prenatal stress and behavioral outcomes [26].

Neurodevelopmental alterations: Prenatal stress can profoundly affect the formation and connectivity of neural circuits crucial for behavior and cognition. These structural alterations can disrupt the normal development of brain regions involved in emotional regulation and cognitive processing, contributing to the emergence of behavioral outcomes observed in offspring [27].

Inflammatory processes: Maternal stress during pregnancy can trigger an inflammatory response, releasing proinflammatory cytokines and other immune molecules. This maternal inflammation can cross the placenta and impact the developing fetal brain. Inflammation during critical periods of brain development has been associated with alterations in neural circuitry and subsequent behavioral changes in offspring [28].

Brain structure and function changes: Prenatal stress can influence the development of specific brain regions and the functioning of neurotransmitter systems. Brain structure and function changes can directly affect behavior and emotional regulation. For example, alterations in the amygdala, prefrontal cortex, and other brain regions critical for emotion processing can contribute to emotional dysregulation and mood disorders [29].

Prenatal stress and cognition

Cognitive Outcomes in Children

Cognitive development milestones: Maternal stress during pregnancy can exert discernible influences on a child's attainment of cognitive development milestones. Research has unveiled associations between prenatal stress exposure and delays in reaching key cognitive milestones. This includes language development, motor skills, and other critical aspects of cognitive growth. These delays indicate disturbances in the intricate process of neural maturation and can resonate throughout a child's developmental trajectory, potentially affecting their overall cognitive development [30].

Academic achievement: The repercussions of prenatal stress extend to the educational domain, where variations in academic achievement come into focus. Children who experience prenatal stress may encounter academic challenges, manifested as lower test scores and diminished academic performance. These challenges can persist across the educational continuum, potentially influencing future academic opportunities and life prospects. The intricate interplay between prenatal stress and academic achievement underscores the enduring impact of early-life experiences on cognitive outcomes [31].

Cognitive deficits and disorders: Prenatal stress is a recognized contributor to an increased risk of cognitive deficits and disorders in children. This risk encompasses a range of conditions, including learning disabilities, intellectual disabilities, and cognitive impairments. The mechanisms that underlie these associations are intricate and multifaceted, involving disruptions in neural circuitry, alterations in brain development, and potential epigenetic modifications. These cognitive deficits and disorders indicate prenatal stress's profound and lasting impact on cognitive functioning, emphasizing the significance of addressing prenatal stress as a critical determinant of cognitive outcomes [32].

Neurobiological Mechanisms Involved in Prenatal Stress and Cognition

Hippocampal changes: Prenatal stress can affect the development and function of the hippocampus, a crucial brain region responsible for learning and memory processes. Altered hippocampal structure and function may contribute to cognitive deficits observed in individuals exposed to prenatal stress. These changes can disrupt the formation and retrieval of memories, influencing an individual's ability to acquire and retain information [33].

Neurotransmitter systems: Prenatal stress can modulate neurotransmitter systems within the brain, including serotonin and dopamine. These alterations can have far-reaching consequences, potentially disrupting cognitive processes related to reward, motivation, and attention. Dysregulation of these systems may result in difficulty maintaining focus, experiencing pleasure, and remaining motivated in cognitive tasks [34].

Synaptic plasticity: The ability of neurons to form and modify connections, known as synaptic plasticity, is integral to learning and memory processes. Prenatal stress may disrupt this crucial neurobiological mechanism, impairing the brain's capacity to adapt and learn. Impaired synaptic plasticity can hinder the formation of new memories and the retention of acquired knowledge, contributing to cognitive deficits [35].

Inflammatory pathways: Prenatal stress can incite inflammatory responses within the maternal and fetal systems. These inflammatory responses adversely affect the developing brain, potentially leading to cognitive impairments. Chronic inflammation can interfere with neural circuitry and disrupt the delicate balance of neurotransmitters, compromising cognitive function [36].

Epigenetic modifications: Epigenetic changes resulting from prenatal stress add another layer of complexity to the neurobiological mechanisms at play. These changes involve modifications to the structure of DNA and histones, influencing the expression of genes involved in cognitive function. Such modifications may persist throughout an individual's life, contributing to cognitive outcomes and potentially increasing the risk of cognitive disorders [37].

Prenatal stress and psychopathology

Psychopathological Outcomes in Offspring

Anxiety disorders: Prenatal stress is closely associated with an elevated risk of anxiety disorders in children and adults. Exposure to heightened levels of maternal stress hormones during pregnancy can influence the development of neural circuits responsible for anxiety regulation. These alterations in neural circuitry can predispose individuals to anxiety-related conditions, shaping their susceptibility to anxious thoughts, behaviors, and disorders throughout life [38].

Mood disorders: The specter of mood disorders, including depression, looms larger for individuals exposed to prenatal stress. Research has unveiled links between prenatal stress exposure, an increased susceptibility to depressive episodes, and a higher risk of developing mood disorders later in life. The mechanisms underpinning these outcomes are multifaceted, involving dysregulation of stress response systems and alterations in brain regions intricately involved in mood regulation. These changes can set the stage for a lifelong vulnerability to mood disorders [9].

Schizophrenia and psychosis: Emerging evidence suggests that prenatal stress may contribute to the risk of schizophrenia and other psychotic disorders. While the exact mechanisms remain a subject of ongoing investigation, disruptions in neural development, alterations in neurotransmitter systems, and immune responses have been proposed as potential contributors to these psychopathological outcomes. Prenatal stress may leave lasting alterations in the brain's structure and function, predisposing individuals to the complex manifestations of schizophrenia and psychosis later in life [39].

Genetic and Environmental Interactions

Genetic variations: The genetic makeup of individuals plays a critical role in modulating their response to prenatal stress. Certain genetic variations can increase or decrease the likelihood of developing psychopathological conditions in response to prenatal stress. For example, specific genetic polymorphisms may render individuals more resilient to the adverse effects of prenatal stress, mitigating their risk of developing anxiety disorders, mood disorders, or other psychopathological outcomes. Conversely, genetic predispositions may amplify the impact of prenatal stress, heightening susceptibility to these conditions [4].

Environmental factors: Environmental factors, particularly those encountered during the postnatal period and early life, further sculpt an individual's risk for psychopathology following prenatal stress. The quality of postnatal caregiving, exposure to nurturing or adverse environments, and the presence of supportive social networks can significantly influence an individual's resilience or vulnerability to psychopathological outcomes. A nurturing and supportive environment can act as a protective buffer, ameliorating the potential consequences of prenatal stress. Conversely, adverse postnatal environments may exacerbate the impact of prenatal stress, amplifying the risk of psychopathology [40].

Moderating factors

Maternal Factors

Maternal stress levels: The severity and duration of maternal stress during pregnancy profoundly influence its impact on offspring. Higher maternal stress levels, particularly chronic stress, may pose a greater risk for adverse outcomes in children. Understanding the dose-response relationship between maternal stress and outcomes is crucial for identifying vulnerable populations and designing targeted interventions. It highlights the need for support for pregnant individuals with high stress levels [41].

Maternal mental health: Maternal mental health is a critical mediator in the relationship between prenatal stress and its effects on offspring. Maternal depression, anxiety, and other psychiatric conditions can exacerbate the impact of prenatal stress on offspring. Moreover, the interaction between maternal stress and mental health can have cascading effects on the developing fetus. Recognizing the interplay between maternal stress and mental health underscores the necessity of comprehensive prenatal mental health support as part of prenatal care [5].

Maternal nutrition and substance use: Maternal nutrition and substance use during pregnancy can significantly modulate the effects of prenatal stress. Inadequate maternal nutrition or exposure to harmful substances such as tobacco, alcohol, or illicit drugs can interact synergistically with prenatal stress, heightening the risk of adverse outcomes in offspring. These factors underscore the critical importance of comprehensive prenatal care that addresses stress management, maternal nutrition, and substance use cessation support [42].

Environmental Factors

SES: SES is a critical environmental factor that can influence the effects of prenatal stress. Individuals from lower SES backgrounds may face additional stressors related to financial instability, limited access to healthcare, and neighborhood disadvantage. These stressors can interact with prenatal stress to magnify its impact on maternal and fetal well-being [43].

Social support: Adequate social support during pregnancy can act as a protective factor against the negative effects of prenatal stress. Strong social networks, including support from family, friends, and healthcare providers, can help buffer expectant mothers' psychological and physiological stress responses. Social support may mitigate the impact of prenatal stress on offspring outcomes [44].

Early-life adversity: Prenatal stress should be considered within the broader context of early-life adversity. Individuals who experience prenatal stress may also encounter postnatal stressors and adverse childhood experiences that further shape their developmental trajectories. Understanding the cumulative impact of early-life adversity is critical for a comprehensive assessment of outcomes [45].

Mechanisms and pathways

Epigenetic Modifications

DNA methylation: Prenatal stress can initiate epigenetic changes, including DNA methylation modifications. DNA methylation involves adding methyl groups to specific genes, which can profoundly influence gene expression patterns. Prenatal stress-induced alterations in DNA methylation may persist into adulthood, carrying the potential to exert enduring effects on an individual's stress regulation, neurodevelopment, and mental health. By modifying the functioning of genes implicated in these critical domains, DNA methylation changes serve as a lasting imprint of prenatal stress on the epigenetic landscape [46].

Histone modifications: Another facet of epigenetic regulation pertains to histone modifications, which prenatal stress can influence. Changes in histone acetylation and methylation patterns can intricately modulate chromatin structure and gene expression. In response to prenatal stress, these modifications can reshape the epigenetic landscape within the developing brain. By influencing the accessibility of genes involved in neurodevelopment and behavior, histone modifications play a pivotal role in potentially contributing to long-term alterations in brain function and behavior observed in individuals exposed to prenatal stress [47].

HPA Axis Dysregulation

Prenatal stress can precipitate the dysregulation of the HPA axis, a cornerstone of the body's response to stress. This dysregulation can manifest as chronic elevations in stress hormones, notably cortisol. Such persistent elevation of cortisol levels can have far-reaching consequences, potentially affecting the developing fetal brain [48].

As the fetal brain is exquisitely sensitive to the influence of cortisol, dysregulated HPA axis function during prenatal stress may contribute to alterations in stress reactivity and vulnerability to mood disorders in adulthood. The developing brain, exposed to prolonged cortisol elevations, may undergo structural and functional changes that impact an individual's lifelong stress response. This altered stress reactivity can set the stage for heightened susceptibility to mood disorders such as depression and anxiety in later life [49].

Understanding the role of HPA axis dysregulation in the prenatal stress paradigm underscores the intricate interplay between early-life experiences and stress response systems. It highlights how disruptions in the HPA axis can reverberate across the lifespan, shaping an individual's vulnerability to mood disorders. Recognizing this mechanism prompts us to explore innovative strategies for mitigating the impact of prenatal stress on the developing HPA axis, with the ultimate goal of fostering healthier stress responses and mental well-being [50].

Neurodevelopmental Alterations

Prenatal stress can exert profound effects on the neurodevelopment of the fetus. This influence extends to various critical processes, including neuronal proliferation, migration, and synaptic connectivity. Disruptions in these neurodevelopmental processes can reverberate across the developing brain, potentially leading to a cascade of consequences [51].

Altered neuronal proliferation can influence the number of neurons available within specific brain regions, with potential implications for cognitive functioning and behavior. Aberrant neuronal migration can result in the misplacement of neurons, disrupting the formation of neural circuits that underlie cognitive processes and emotional regulation. Changes in synaptic connectivity can further compound these alterations, affecting the efficiency of neural communication [52].

These disruptions in neurodevelopmental processes can collectively impact brain structure and function, potentially contributing to cognitive deficits, behavioral changes, and the development of psychopathology. The intricate interplay between prenatal stress and neurodevelopment underscores the lasting consequences of early-life experiences on the structure and function of the brain, highlighting the importance of early intervention and support to mitigate these potential effects [53].

Inflammatory Processes

Maternal stress during pregnancy can trigger inflammatory responses within the maternal and fetal systems. These responses result in elevated levels of proinflammatory cytokines and immune molecules, creating an inflammatory milieu within the maternal body [54]. Notably, this inflammation does not remain confined to the maternal domain. Proinflammatory molecules can cross the placenta, breaching the fetal sanctuary and infiltrating the developing fetal brain.

The implications of inflammation within the fetal brain are multifaceted [55]. Firstly, inflammation during prenatal development may disrupt the intricate process of neural development, potentially leading to structural and functional alterations in the brain. These disruptions can have far-reaching consequences for cognitive functioning and behavior [56]. Secondly, increased susceptibility to neuroinflammatory conditions may result from prenatal stress-induced inflammation. The fetal brain, exposed to heightened inflammatory signals, may be more vulnerable to neuroinflammatory diseases later in life [57]. Lastly, behavioral alterations may stem from prenatal stress's neuroinflammatory effects. Inflammation within the developing brain can influence neural circuits associated with emotional regulation and behavior, potentially contributing to the emergence of behavioral changes and psychopathological outcomes in offspring [40].

Brain Structure and Function Changes

Amygdala: The amygdala, a hub for emotional processing, can undergo structural and functional modifications in response to prenatal stress. These alterations may result in heightened emotional reactivity, difficulties in emotional regulation, and an increased susceptibility to anxiety-related conditions. Changes in the amygdala's size and connectivity can shape an individual's emotional landscape, contributing to the emergence of behavioral outcomes related to emotional dysregulation [58].

Prefrontal cortex: The prefrontal cortex, responsible for higher-order cognitive functions and executive control, may also be affected by prenatal stress. Alterations in prefrontal cortex structure and function can disrupt cognitive processes such as decision-making, impulse control, and attention. These changes can manifest as cognitive deficits and challenges in managing complex tasks, influencing academic achievement and overall cognitive functioning [59].

Hippocampus: The hippocampus, a central player in memory formation and stress regulation, is not immune to the effects of prenatal stress. Structural and functional changes in the hippocampus can impact an individual's ability to encode, consolidate, and retrieve memories. These alterations may contribute to cognitive deficits, impairments in learning, and difficulties in coping with stressors [60].

Intervention and prevention strategies

Prenatal Stress Reduction Programs

Psychoeducation: Prenatal stress reduction programs often incorporate psychoeducational components as a cornerstone of their approach. These programs are designed to equip expectant mothers with essential knowledge about the effects of stress during pregnancy. By understanding the impact of stress on maternal and fetal health, participants can make informed choices and adopt strategies for effective stress management. Psychoeducation includes teaching relaxation techniques, mindfulness practices, and stress-reduction exercises. It empowers expectant mothers to recognize and navigate the stressors in their lives, promoting emotional well-being and resilience [61].

Mind-body interventions: Mind-body interventions constitute a valuable dimension of prenatal stress reduction programs. Yoga, meditation, and tai chi have effectively mitigated prenatal stress. These mind-body techniques foster relaxation, alleviate anxiety, and enhance emotional well-being. Incorporating mind-body interventions into prenatal care benefits maternal mental health and can positively affect fetal development. By promoting a sense of calm and emotional balance, these practices contribute to a healthier prenatal environment [62].

Maternal Mental Health Support

Counseling and therapy: Expectant mothers grappling with significant prenatal stress can benefit immensely from counseling and therapy services. Evidence-based therapeutic approaches such as cognitive-behavioral therapy (CBT) offer a structured framework for addressing stress-related challenges. Through counseling and therapy, mothers can develop effective coping strategies, manage symptoms of anxiety and depression, and nurture their overall mental well-being. These interventions empower expectant mothers to navigate the emotional complexities of pregnancy and build resilience [63].

Perinatal mental health services: Establishing accessible perinatal mental health services is instrumental in supporting expectant mothers. These services should encompass a comprehensive approach, starting with routine screening for mental health issues during prenatal care. Counseling or therapy should be readily available for those in need, providing a safe space to address stressors and emotional concerns. Furthermore, these services should extend into the postpartum period to ensure ongoing support and continuity of care. By integrating perinatal mental health services into the standard of care, healthcare systems can proactively identify and address maternal mental health needs [64].

Nutritional Interventions

Balanced diet and supplements: The significance of a balanced diet during pregnancy cannot be overstated, as it serves as the cornerstone of both maternal and fetal health. Ensuring expectant mothers receive a diet rich in essential nutrients provides vital support for the developing fetus. Nutrients like folic acid, iron, and calcium are essential for proper fetal growth and development. When dietary intake is insufficient, supplements such as prenatal vitamins can bridge nutritional gaps. These supplements offer a convenient and reliable means of delivering the necessary vitamins and minerals, bolstering healthy pregnancy outcomes [65].

Omega-3 fatty acids: Omega-3 fatty acids, with a particular focus on docosahexaenoic acid (DHA), have garnered attention for their potential to counteract the effects of prenatal stress. DHA, integral to brain development, holds promise as a protective agent against the impact of stress on the developing fetus. Ensuring an adequate intake of omega-3 fatty acids through dietary sources like fatty fish or supplements may contribute to healthier fetal brain development and potentially mitigate the adverse consequences of prenatal stress [66].

Targeted Early Interventions for At-Risk Populations

Identifying at-risk populations: Early identification of at-risk populations is the linchpin of effective intervention. This includes individuals with a history of trauma, high levels of stress during pregnancy, or other risk factors. By recognizing these vulnerable groups early in the prenatal journey, healthcare providers can tailor interventions to meet their needs. Personalized approaches ensure that resources and support are directed where they are most needed [67].

Home visitation programs: Home visitation programs, facilitated by trained professionals or community health workers, offer a direct and holistic approach to support at-risk families. These programs extend a helping hand to expectant mothers and families within the comfort of their homes. Home visitors provide help in stress management, parenting practices, and accessing essential resources. By establishing a trusted and supportive relationship, these programs empower families to navigate the challenges of prenatal stress effectively. They bridge at-risk populations and the healthcare and social support systems [68].

Early childhood interventions: Early interventions that commence during infancy or early childhood hold the potential to counteract the long-term effects of prenatal stress. These interventions encompass a spectrum of initiatives, including early childhood education programs, social-emotional development support, and parenting education. By focusing on the critical early years of a child's life, these interventions promote healthy development, enhance resilience, and mitigate the impact of prenatal stress. They equip children with essential skills and support systems to thrive despite early-life adversity [69].

Future directions and research gaps

Emerging Areas of Research

Multiomics approaches: Emerging research incorporates multiomics approaches, combining genomics, epigenomics, transcriptomics, proteomics, and metabolomics to provide a more comprehensive understanding of the molecular mechanisms underlying prenatal stress effects. These approaches enable researchers to uncover complex interactions among genetic, epigenetic, and environmental factors [70].

Microbiome-brain axis: Investigating the gut-brain axis and the role of the maternal and fetal microbiome in prenatal stress is an emerging area of interest. It explores how the gut microbiome might influence neurodevelopment and mental health outcomes in offspring and how prenatal stress can impact the maternal microbiome [71].

Long-term multigenerational effects: Research is beginning to explore the potential multigenerational effects of prenatal stress. Understanding how prenatal stress experienced by one generation may impact the health and development of subsequent generations is a complex yet intriguing area of investigation [72].

Methodological Improvements Needed

Standardized measurement of prenatal stress: The field would benefit from standardized measures of prenatal stress to ensure consistency and comparability across studies. This includes developing validated tools for assessing prenatal stress levels, including subjective and objective measures [73].

Longitudinal and multimodal approaches: There is a need for more longitudinal studies that follow individuals from prenatal development through adulthood. Additionally, integrating multimodal assessments, including neuroimaging, genetics, and psychophysiology, can provide a more comprehensive understanding of the mechanisms and trajectories associated with prenatal stress [74].

Causal inference: Establishing causality in the relationship between prenatal stress and outcomes is challenging due to ethical constraints and confounding variables. Advancements in research designs, such as natural experiments and Mendelian randomization, can help address this issue [75].

Potential Translational Applications

Precision medicine: Tailoring interventions based on an individual's genetic and epigenetic profile and prenatal stress exposure holds promise for precision medicine approaches. Identifying biomarkers that predict vulnerability to prenatal stress-related outcomes can guide personalized interventions [76].

Prenatal care integration: Integrating prenatal stress assessment and intervention into routine prenatal care can be a powerful strategy for preventing adverse outcomes. Healthcare providers can play a pivotal role in identifying at-risk individuals and offering appropriate support [77].

Public health initiatives: Public health campaigns can raise awareness about the importance of prenatal mental health and stress reduction. Strategies to reduce stress among pregnant individuals, such as promoting mindfulness and stress-reduction programs, can be incorporated into community-based initiatives [78].

## Conclusions

This comprehensive review has shed light on the profound and lasting impact of prenatal stress on behavior, cognition, and psychopathology. The key findings underscore the significance of understanding the intricate interplay between maternal stress during pregnancy and its consequences for offspring. Prenatal stress is not merely a transient experience but a critical determinant of an individual's lifelong well-being. This knowledge carries important implications for both public health and clinical practice. It emphasizes the urgent need to prioritize maternal mental health, implement evidence-based interventions, and raise awareness about the lasting effects of prenatal stress. By addressing this critical issue, we can work towards a future where expectant mothers and their children thrive, and the burden of mental health disorders is reduced, leading to healthier generations and stronger communities.
